# Determining the Impact of a Physiotherapist-Led Primary Care Model for Hip and Knee Pain: Protocol and Analysis Plan for a Cluster Randomized Controlled Trial With Process Evaluation

**DOI:** 10.2196/89006

**Published:** 2026-05-22

**Authors:** Jordan Miller, Catherine Donnelly, Chad McClintock, Kevin Varette, Yeimi Camargo, Jacquelyn Marsh, Monica Taljaard, Mir Sanim Al Mamun, Geneviève Bacchus, David Barber, Lynn Cooper, Simon French, Michael Green, Jonathan Hill, Joy MacDermid, Kathleen Norman, Julie Richardson, Joan Tranmer, Timothy Wideman

**Affiliations:** 1 School of Rehabilitation Therapy Faculty of Health Sciences Queen's University Kingston, ON Canada; 2 Health Services and Policy Research Institute Faculty of Health Sciences Queen's University Kingston, ON Canada; 3 School of Physical Therapy Western University London, ON Canada; 4 Ottawa Hospital Research Institute Ottawa, ON Canada; 5 School of Epidemiology and Public Health University of Ottawa Ottawa, ON Canada; 6 Department of Family Medicine Faculty of Health Sciences Queen's University Kingston, ON Canada; 7 Spinal Pain Research Centre Department of Chiropractic Macquarie University Sydney, New South Wales Australia; 8 NOSM University Thunder Bay, ON Canada; 9 School of Medicine Keele University Newcastle-underLyme, England United Kingdom; 10 School of Rehabilitation Science McMaster University Hamilton, ON Canada; 11 School of Nursing Queen's University Kingston, ON Canada; 12 School of Physical and Occupational Therapy McGill University Montreal, QC Canada

**Keywords:** cluster randomized trial, health services research, physiotherapy, primary care, process evaluation

## Abstract

**Background:**

Hip and knee pain are leading contributors to disability, reduced quality of life, and health care burden in Canada. Primary care is often the first point of contact for patients with these conditions, yet timely and appropriate care is limited due to provider shortages and system pressures. Interest is growing in interprofessional primary care models that integrate physiotherapists to enhance care delivery for musculoskeletal conditions such as hip and knee pain.

**Objective:**

This study aims to communicate the protocol and analysis plan for a trial with two objectives: (1) to evaluate the effectiveness of a physiotherapist-led primary care model for hip and knee pain on physical functioning (primary outcome), pain intensity, quality of life, global rating of change, patient satisfaction, and adverse events, compared to usual physician-led primary care; and (2) to assess the impact of this model on health care system and societal outcomes, including access to care, health care use, productivity loss, and cost-effectiveness. A process evaluation will examine implementation, potential mechanisms, and patient experiences.

**Methods:**

A cluster randomized controlled trial involving 14 primary care organizations randomized equally to either a physiotherapist-led or usual physician-led primary care model for patients with hip and knee pain. Patients were recruited over 1 year, with data collected at baseline and at 3, 6, 9, and 12 months. The intervention integrates a physiotherapist as the initial point of contact within the primary care team for patients seeking care for hip or knee pain. It includes four components: (1) comprehensive assessment and screening, (2) brief individualized interventions during the initial visit, (3) guidance for accessing additional health care resources, and (4) follow-up physiotherapy for patients with unmet needs. Effectiveness will be assessed using linear mixed regression, accounting for clusters and prespecified covariates. The multimethods process evaluation will include descriptive and comparative analysis of implementation, mediation analysis to explore potential mechanisms, and qualitative exploration of patient experiences.

**Results:**

This research was funded in December 2022. Primary care sites (clusters) were recruited and randomized in June and July 2023, respectively. Patient enrollment occurred from October 2023 through November 2024. The final patient follow-up survey was completed in November 2025. Extraction of data from electronic health records is expected to finish in December 2025. Data analysis will begin after data collection is complete and will follow the predefined protocol and analysis plan. No interim analyses are planned.

**Conclusions:**

Findings from this trial will provide actionable evidence on whether integrating physiotherapists into primary care teams for hip and knee pain improves patient outcomes and health care system efficiency. Effectiveness and process evaluation evidence will inform policymakers and health system leaders on the adoption and implementation of interprofessional, team-based primary care models.

**Trial Registration:**

ClinicalTrials.gov NCT06358521; https://clinicaltrials.gov/study/NCT06358521

**International Registered Report Identifier (IRRID):**

DERR1-10.2196/89006

## Introduction

### Overview

Hip and knee pain are leading contributors to reduced functioning. Osteoarthritis alone is one of the leading causes of pain, disability, and reduced quality of life [1], and the hip and knee are the most common body regions [2,3]. Arthritis currently affects 1 in 5 Canadians, and the prevalence is expected to rise [2]. Osteoarthritis places a substantial burden on society in terms of both direct and indirect costs, including reduced work productivity and missed work [2,3].

Hip and knee pain and other musculoskeletal conditions are among the most common reasons for a patient to access primary care [4-6]. Due to the rise in patients seeking support at the primary care level for many chronic conditions and a growing shortage of primary care providers, patients often do not receive timely access to the care they require [7-10]. Additionally, for patients without primary care providers, their first point of contact for their pain is often the emergency department (ED), which contributes to long wait times and overcrowding of the EDs [11].

There is an urgent need for evidence-informed and patient-centered interprofessional primary care models to meet the needs of patients with hip and knee pain. In Canada, federal and provincial governments have identified that interprofessional teams with complementary skillsets are required to address patients’ multiple needs and to improve the effectiveness, efficiency, and sustainability of the health care system [12,13]. Research from other health conditions suggests that team-based primary care can improve access to appropriate care, coordination of care, and patient outcomes [14-16]. One example of such an integrated model of care is having a physiotherapist [17] integrated within primary care teams and available as the first point of contact (physiotherapist-led primary care). Physiotherapists can provide comprehensive and efficient management of patients seeking primary care for musculoskeletal conditions such as hip and knee pain, and evidence suggests that physiotherapists are able to provide collaborative care in a primary care setting [18-21].

Previous studies on physiotherapists working in primary care for musculoskeletal disorders have demonstrated that physiotherapists provide equal or improved care compared to physicians or nurse practitioners and that patient satisfaction is high when being managed by a physiotherapist [22-24]. Studies conducted in the United Kingdom concluded that physiotherapists working in primary care resulted in freeing up primary care practitioners’ time, reduced referrals to secondary and tertiary care, fewer requests for diagnostic imaging, increased patient satisfaction, and potential for cost savings [25-28]; however, existing evidence is limited to primarily observational studies or small trials, providing low to very low quality of evidence on effectiveness [29].

The impact of a physiotherapist-led primary care model for patients with hip and knee pain has not been examined in the Canadian context. High-quality evidence is needed to assess the effects of the physiotherapist-led primary care model on the following: patient health outcomes, access to care, health service use, and society (eg, occupational productivity and costs). Additionally, there is a need to assess how this model of care is implemented, potential mechanisms of the model, and patients’ experiences with the model of care within the Canadian health system context in order to inform the adoption and implementation of this model of care, if effective.

The goal of publishing this protocol and analysis plan for our cluster trial and embedded process evaluation is to transparently communicate our design and methods in enough detail to be reproduced and to communicate the analytic plan in advance of analysis to reduce the risk of analytic or reporting bias.

### Research Objectives

The study has the following two research objectives:

1. To determine the effectiveness of a physiotherapist-led primary care model for people with hip and knee pain at improving function (primary outcome), pain intensity, quality of life, global rating of change, patient satisfaction, and adverse events compared to usual physician-led primary care, when evaluated over a 1-year period from the initial consultation.
2. To assess the impact of a physiotherapist-led primary care model for patients with hip and knee pain on the health system and society (health care access, primary care physician workload, health care use, missed work, and cost-effectiveness), evaluated over a 1-year period from initial consultation.

## Methods

### Design

**Figure 1:**
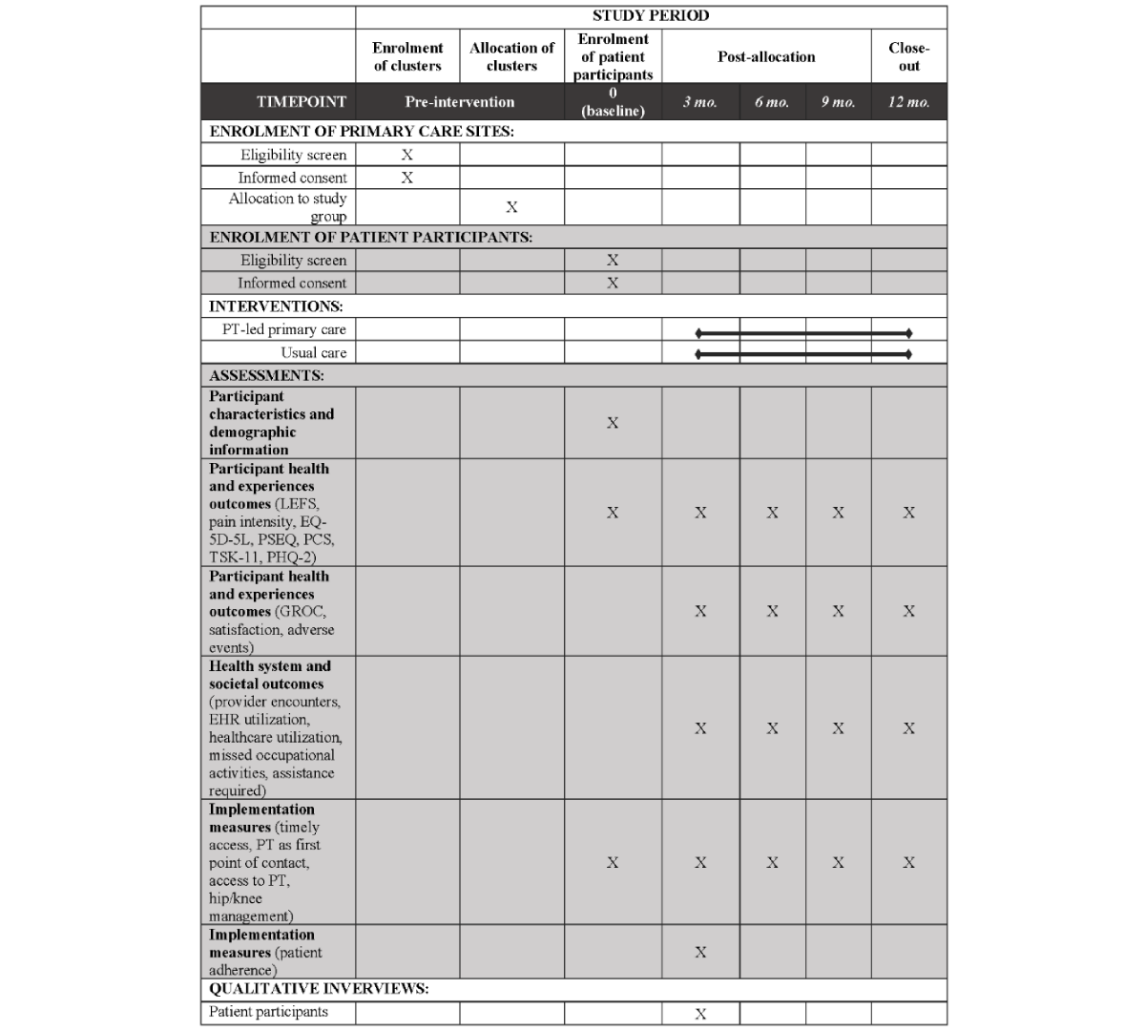
Schedule of enrollment, interventions, and assessments. EHR: electronic health record; GROC: global rating of change; LEFS: Lower Extremity Functional Scale; PCS: Pain Catastrophizing Scale; PHQ-2: 2-item Patient Health Questionnaire; PSEQ: Pain Self-Efficacy Questionnaire; TSK-11: Tampa Scale of Kinesiophobia-11.

The trial is a parallel arm cluster randomized controlled trial (RCT) conducted across 14 primary care sites in Ontario, Canada. Participating sites were randomized 1:1 to either a physiotherapist-led primary care model or the usual physician-led primary care model for hip and knee pain. Randomizing at the practice level, rather than the patient level, enabled full integration of physiotherapists within the primary care team and minimized the risk of contamination between providers [30]. This protocol and analysis plan is according to the SPIRIT (Standard Protocol Items: Recommendations for Interventional Trial) reporting guidelines [31,32]. See Figure 1 for an overview of the schedule of enrollment, interventions, and assessments as recommended in the SPIRIT guidelines.

The trial includes a multimethods process evaluation, guided by the United Kingdom Medical Research Council (MRC) framework for developing and evaluating complex interventions [33,34]. The process evaluation is exploring how the intervention is being implemented, differences in primary care provided to people with hip and knee pain receive in the physiotherapist-led primary care model in comparison to the usual care model, potential mechanisms of the model of care, and experiences of patients with this model of care. This is essential for interpreting trial results; for example, understanding why the intervention may fail, yield unintended effects, or succeed, and can help guide future implementation of this model of care in the future if effective [34,35].

This cluster randomized trial is being run alongside another cluster randomized trial (clinicalTrials.gov NCT04287413) that aims to determine the impact of a physiotherapist-led primary care model for people with low back pain. The protocol and analysis plan presented here is for a trial focused on hip and knee pain being carried out at 14 of the 20 sites participating in the low back pain trial (14 sites located in Ontario are included, 6 sites in British Columbia are not included). We used the existing randomization schedule created for the low back pain trial to leverage the physiotherapists and research staff already hired for the low back pain study. The multimethods process evaluation for the low back pain trial includes a mixed methods exploration of how the context influences implementation of a physiotherapist-led primary care model and a qualitative exploration of the perspectives of primary care team members. These elements were intentionally not included or replicated in the process evaluation plan for the hip and knee trial. However, we anticipate findings related to the perspectives of primary care team members and contextual factors influencing implementation of the physiotherapist-led primary care model explored as part of the process evaluation in the low back pain trial will be informed by experiences implementing a physiotherapist-led primary care model for both people with low back pain and people with hip or knee pain. The process evaluation for this hip and knee trial will provide an understanding of how the physiotherapist-led primary care model for patients with hip and knee pain was implemented, explore the potential mechanisms of the interventions (if effective), and capture the experiences of patients who received care through this model.

### Patient and Public Involvement

A person with lived experience was involved throughout this study, contributing to the conceptualization of the study, study design, and selection of outcomes important to people with lived experience. Their ongoing involvement included pretesting data collection tools, supporting the interpretation of process evaluation results, and codeveloping knowledge mobilization supports such as tailored summaries that will be distributed through patient organizations.

### Enrollment and Randomization of Sites

We enrolled all 14 Ontario sites from the 20 sites included in our cluster-RCT, testing a similar model of care for people with low back pain in Ontario and British Columbia. We used the existing randomization schedule [17], with a 1:1 ratio for physiotherapist-led and usual physician-led arms. Covariate constrained randomization [36] was used by an independent statistician to reduce the risk of baseline imbalances across study arms; the number of active patients and rural versus urban clinic setting were used as covariates. Each cluster name was concealed with a deidentified code prior to randomization. The maximum tolerable difference for rural/urban was 1. The strata balancing criteria was 10% for the number of active patients.

### Strategies to Mitigate Bias

Given the nature of the trial design and the models of care being compared, blinding was not feasible for the physiotherapists, patient participants, primary care team members, or research assistants (RAs). While this limitation is inherent to the study design and models of care, we implemented several measures recommended for cluster randomized trials to reduce potential bias [37]. A common concern in cluster RCTs is the risk of selection and recruitment bias, particularly when patient participant enrollment occurs after cluster assignment [38]. To mitigate the risk of selection and recruitment bias, we took efforts to invite consecutive patients with hip or knee pain to participate in the study, to ensure RAs who handled recruitment and consent were not familiar with the patients, and to apply inclusion and exclusion criteria consistently across all sites. We also provided identical trial information to all potential participants before obtaining consent, regardless of cluster. This meant that we did not reveal which study arm participants were in until after obtaining their consent.

### Patient Participant Recruitment

During patient participant recruitment, medical secretaries screened patients to be invited to participate when they booked an appointment for hip or knee pain. Potential participants were also identified by any health care provider (HCP) when they sought care for their hip or knee pain during a primary care encounter. Potential participants who agreed to be invited to participate were contacted by a study RA to explain the details of the study and provide the opportunity for questions. Participants completed an electronic consent form that was built into the baseline surveys in a secure data collection platform (REDCap [Research Electronic Data Capture; Vanderbilt University]). If a potential participant requested to complete the baseline assessment in-person, arrangements were made to meet with the RA, who obtained written informed consent for participation from those who were interested. A verbal consent process was in place for those who requested to complete the baseline assessment virtually.

### Inclusion/Exclusion

All adult (≥19 years) patients who sought primary care for their hip or knee pain of any duration at participating sites were invited to meet with the RA to discuss participating in the study. Potential participants were excluded if they reported not being able to understand, read, and write English; they had known cancer causing their hip or knee pain; or they reported being unable to complete the scheduled follow-up surveys over the 1-year study period. The inclusion criteria for primary care sites were to have at least 1500 rostered patients and 2 or more family physicians. Sites that already had physiotherapy services or did not have enough space to include a physiotherapist were excluded.

### Study Power

Participants were recruited over a 13-month period at all 14 primary care sites (7 physiotherapist-led arm and 7 usual care arm). Our power calculation is based on methods described by Hemming et al [39]. Our sample size calculation is targeted to detect a clinically meaningful mean difference of 9 points on the Lower Extremity Functional Scale (LEFS) using a 2-sided α of .05, assuming a conservative SD of 16, a conservative intracluster correlation coefficient of 0.1, a cluster autocorrelation coefficient of 0.5, an individual autocorrelation coefficient of 0.6, and a coefficient of variation of 0.7 [40-42]. Allowing for an attrition of 20% of patient participants at the 12-month follow-up, we require a sample size of 560 participants to achieve >80% power. Pilot work for this study suggested we would recruit 1 patient participant per week per site; as such, we planned and prepared resources to recruit approximately 728 participants.

### Interventions

#### Physiotherapist-Led Primary Care Model for Hip and Knee Pain

##### Overview

The index intervention is a model of care that incorporates a physiotherapist within the primary care team as an option for the first point of contact for people presenting with hip or knee pain for 1 year from the time of enrollment. Patient participants in this model are given the choice of seeing the physiotherapist or the primary care provider first. There are four key components of the physiotherapist-led model of care: (1) initial assessment and screening, (2) brief individualized care at first visit, (3) health services navigation, and (4) providing additional physiotherapist care for people with an unmet need (eg, no insurance coverage for physiotherapist).

##### Initial Assessment and Screening

The physiotherapist provides a comprehensive patient assessment according to established clinical practice guidelines. The assessment includes taking a detailed clinical history; screening for potential pathology and the need to refer to another HCP; physical examination; and using a validated patient-reported outcome measure (LEFS [40]) to guide clinical decision-making.

##### Brief Individualized Care at the First Visit

The physiotherapist intervention is flexible and determined by the physiotherapist and patient participant to reflect real-world physiotherapist practice and facilitate generalizability. The care provided is intended to align with practice guidelines. This could include education, physical activity advice, exercises, or a referral to another HCP or community program.

##### Health Services Navigation

Where applicable, patient participants are provided with options available to them in their community for rehabilitation programs or other health or social services. For example, they may be referred to a community physiotherapist for ongoing management or presented with options for group exercise programs. Patient participants are assessed regarding the need for specialist referrals or resources available to manage complex clinical presentations such as comorbidity, frailty, or the need for surgical consultation. Patient participants are referred to the primary care provider if specialized services are needed or when the physiotherapist cannot provide a direct referral. In addition, patient participants are referred back to their primary care provider when it is deemed that their clinical needs fall outside of the physiotherapist’s scope of practice (ie, medication advice and pathology requiring medical attention) or they identify factors that require mental health intervention.

##### Providing Additional Physiotherapist Care

Patient participants who require a physiotherapist but do not have the appropriate access to community-based services are managed by the physiotherapist who provided the assessment in the primary care setting. Individuals with private or government health coverage are referred to services outside of the primary care practice when they are accessible to the patient participant in order to avoid duplication of available services. The amount of care provided is decided on a case-by-case basis in alignment with the health needs of each individual.

##### Physiotherapist Training

During 2 consecutive days, 7 registered physiotherapists participated in training on this new model of care in preparation for delivering the model of care across the 7 sites randomized to the physiotherapist-led primary care model for hip or knee pain. The training included education about the primary care context, a review of clinical practice guidelines and best practices for the management of hip and knee pain, and a discussion of how to access health services available for hip and knee pain through referrals.

#### Usual Care Model

The physician- or nurse practitioner–led primary care intervention is unstandardized to best reflect the usual primary care clinical practice in Ontario. Patient participants in the usual care group are seen by either a primary care physician or a nurse practitioner as their first point of contact, depending on the current practice at the clinic. The inclusion of only primary care organizations who did not have a physiotherapist on their team means patient participants in this arm neither receive a primary care assessment from a physiotherapist, nor do they have access to a physiotherapist as a member of their primary care team; however, participants in both groups are permitted to seek additional care from interprofessional team members within their primary care team or health services outside of the primary care clinic, including community physiotherapists, as needed or recommended by the primary care physician or nurse practitioner.

#### Duration of Treatment Period

The intervention was carried out over a 1-year period from the time of consent. All participants in the intervention arm are offered an initial assessment with the physiotherapist and have access to the physiotherapist as a member of their primary care team throughout the 1-year follow-up period if they require additional support for their hip or knee pain. The frequency and duration of visits is determined by the physiotherapist and patient participant. The usual care model is ongoing and involves continuing as usual over the 1-year study period.

#### Intervention Modifications

We do not expect any safety-related issues that would necessitate removing a participant from either the physiotherapist-led or usual physician-led primary care pathway. In line with routine care practices for hip or knee pain, the primary care team will adjust the intervention as needed to prioritize participant well-being. Adjustments may be made in response to factors such as increased pain, limited mobility, poor tolerance to treatment, changes in clinical status, or adverse reactions to medications or exercises. As the risks are minimal, a data safety and monitoring committee was not established.

### Data Collection and Management

#### Overview

All baseline measures have been collected from participants, and follow-up data have been collected. We used several approaches to support participant retention at all time points. RAs sent reminders every 2-3 days via personalized emails, phone calls, and SMS text messages to encourage survey completion. When preferred by participants, surveys were completed in person or by phone to enhance engagement and reduce the likelihood of attrition.

All assessment data at baseline and follow-up time points were collected through online surveys using REDCap [43], a secure online survey and data capture tool, which is hosted at Queen’s University. Electronic health record (EHR) data were extracted by trained RAs at the end of the 1-year intervention period directly into a securely stored database at Queen’s University. Audio recordings from qualitative interviews were transcribed, deidentified, and stored in a secure Microsoft OneDrive environment at Queen’s University. At the end of the study, deidentified survey responses, EHR data, and interview transcripts will be securely stored in Microsoft OneDrive at Queen’s University.

#### Baseline Characteristics Used to Describe the Population

We collected the following baseline information from participants: age, sex, gender, education, duration of hip/knee pain, locations of pain, medications, comorbidities, employment status, income, rurality, and ethnicity. Comorbidities are assessed using the Functional Comorbidity Index [44-46] (a list of comorbidities that are associated with physical functioning). The presence of a comorbidity is assigned a score of 1, and the total score is the sum of the comorbidity elements with a maximum score of 18.

#### Individual Health and Experience Outcomes

##### Overview

The following individual health and experience measures were collected through REDCap with repeat surveys at baseline, 3, 6, 9, and 12 months post enrollment, with a primary time point of comparison at 12 months. Patient satisfaction, global rating of change, and adverse events were collected at all follow-up time points only.

##### Self-Reported Functioning (Primary Outcome)

Self-reported functioning was assessed using the LEFS [40]—a validated 20-item patient-reported outcome measure used to assess functional status related to lower extremity conditions. Each item is scored on a 5-point scale, with higher scores indicating better function.

##### Pain Intensity

Pain intensity was measured using a Numeric Pain Rating Scale (NPRS) [47], where 0 is no pain, and 10 is the worst possible pain.

##### Health-Related Quality of Life

Health-related quality of life was measured using the EQ-5D-5L, which is suitable for economic evaluations [48,49]. The EQ-5D-5L score will also be converted to quality-adjusted life years (QALYs) [48].

##### Global Rating of Change

Global rating of change was assessed using an 11-point global rating of change scale to assess perceived overall change in health status, symptoms, or function over time, with anchors of a great deal better (+5) to a great deal worse (–5) [50].

##### Patient Satisfaction

Patient satisfaction was assessed using an 11-point scale with anchors of very dissatisfied (–5) and very satisfied (+5).

##### Adverse Events

Adverse events were measured using an adverse events questionnaire aligned with reporting guidelines [27,28]. The questionnaire determines (1) adverse events experienced as a result of any of the interventions received, (2) a description of the adverse event, (3) duration of the adverse event, and (4) severity of the adverse event. Serious adverse events are identified if the participant requires hospitalization or an ED as a result of the adverse event, the adverse event leads to significant and persistent disability beyond 72 hours, or the adverse event is life-threatening. The study team monitored these responses to ensure ongoing patient participant safety through an automated alert system within REDCap that sent an automatic email notification to the study team, who promptly reviewed the responses.

#### Potential Mechanisms of the Intervention

##### Self-Efficacy

Self-efficacy is the confidence in abilities to participate in usual activities, which was assessed using the Pain Self-Efficacy Questionnaire (PSEQ) [51,52].

##### Psychosocial Risk Factors for Persistent Pain and Disability

Psychosocial factors associated with pain-related disability were measured using the Pain Catastrophizing Scale (PCS) [53-55], Tampa Scale of Kinesiophobia-11 (TSK-11) [56-58], and 2-item Patient Health Questionnaire (PHQ-2) [59].

#### Health System and Societal Outcomes

##### Primary Care Physician or Nurse Practitioner Visits

Visits to a primary care physician or nurse practitioner refer to the total number of patient participant visits, both initial and follow-up, related to hip or knee pain. This metric was used to explore whether involving physiotherapists in care delivery helps alleviate demand on primary care providers, potentially allowing them to allocate more time to patients with other health concerns.

##### Health Care Use Within the Primary Care Team

Health care use within the primary care team is defined as consultations with all primary care team members (eg, physicians, nurse practitioners, nurses, social workers, and occupational therapists), including physiotherapist at intervention sites, as well as participation in group programs offered within the organization. These data were collected from the EHR abstraction process.

##### Health Care Use Outside of the Primary Care Team

Health care use outside of the primary care team refers to medications used; walk-in clinic visits; ED visits; inpatient hospital stays; diagnostic imaging; surgeries, injections, and other interventional procedures; visits to specialist physicians; and visits to other health professionals outside the primary care team (eg, chiropractors, massage therapists, occupational therapists, physiotherapists, and chronic pain clinics). These outcomes were collected from the self-report surveys at each follow-up assessment and verified, whenever possible, in the EHR.

##### Missed Occupational Activities

Missed occupational activities include self-reported time lost from paid employment, volunteer, homemaking, or educational activities.

##### Assistance Required

Assistance required is defined as the self-reported paid and unpaid assistance required. For example, self-care (eg, taking medications, dressing/undressing, going to the bathroom, bathing/showering, and grooming), shopping/groceries, meal preparation, housework, managing finances, or transportation (eg, to a medical appointment).

##### Costs

Total per-person costs include both direct and indirect health care costs, with indirect costs estimated using a human capital approach based on time missed from work or other daily activities [60].

Direct costs incorporate intervention-related expenses (eg, physiotherapist salary and training), publicly funded health care services (sourced from the Ontario Ministry of Health Schedule of Benefits [61]), medication costs (using the Ontario Drug Benefit formulary), and participant-reported expenses for privately funded services or out-of-pocket supports (eg, self-care, household help, and transportation). Resource use will be multiplied by relevant unit costs to estimate total expenditures, which will be summed over each follow-up interval and used to calculate both time-specific and overall costs.

Indirect costs reflect productivity losses due to time away from paid work, valued using the provincial average wage from Statistics Canada for participants not engaged in paid employment (eg, retirees, homemakers, caregivers, and students). Lost time from unpaid activities will be valued using the minimum wage in Ontario.

#### Implementation Measures

Consistent with the MRC guidelines [34], we collected the following implementation measures as part of our process evaluation:

Timely access to care: determined by the percentage of patient participants with hip or knee pain who are assessed within 48 hours of calling for an appointment. Only participants who were invited to participate at the time of calling for an appointment for their hip or knee pain will be included in this analysis. Participants who were invited to participate at the time of an appointment with another primary care provider will not be included.

First contact care by the physiotherapist: using the percentage of patient participants with hip or knee pain in the physiotherapist-led primary care arm who visited a physiotherapist as their first point of contact for the current episode of hip or knee pain.

Hip and knee pain management provided: visits to the primary care site related to hip and knee pain were collected from the EHR, along with indicators for the following process measures: education provided; exercises prescribed; psychological interventions provided; referrals made to internal primary care team members; referrals made to external HCPs; medications prescribed, deprescribed, and suggested; diagnostic imaging ordered; notes sent to employers or insurers; messages sent to internal primary care team members; and other interventions provided. 

Patient adherence to recommendations: adherence to physiotherapist activity and exercise recommendations was collected at the 3-month follow-up survey. We will also be able to determine if participants accessed a community physiotherapist as part of each follow-up survey time point.

#### Qualitative Interviews

Qualitative interviews were conducted with patient participants following an interpretive description approach [62] to explore their experiences with the physiotherapist-led model of care for hip and knee pain, and their perspectives toward the model of care. A purposive sampling strategy was used [63]. We aimed to recruit 8-12 patient participants representing diversity in age, gender, race, income, employment status, pain duration and intensity, baseline function, and primary care clinic. We are using the concept of information power [64] to determine sample size adequacy within each group—ending recruitment once sufficient depth and breadth of data have been obtained to meet the study objectives. During the consenting process for the main part of the study, patient participants were asked if they were willing to be contacted for qualitative interviews exploring their experiences and perspectives of the physiotherapist-led model of care. Using the purposive sampling approach, agreeable patient participants were contacted, approximately 2-3 months after enrollment, to explain the purpose of the interviews, discuss the consent process and letter of information, and schedule an interview time. At the beginning of the interview, the researcher completing the interview confirms that the participant has read the consent form and answers any questions they may have before obtaining verbal consent.

#### Protocol Amendments

Any protocol modifications will be documented through updates to the ClinicalTrials.gov registry and described in the final trial publication. Investigators and participants will be informed as needed, depending on the nature of the changes.

#### Data Analysis Plan

##### Effectiveness Analyses

All analyses will be conducted by the intention-to-treat principle. Descriptive statistics will be provided for baseline characteristics and outcomes using means (SDs) or medians (IQRs) for continuous variables and frequencies (percent) for categorical variables. We will compare arms using linear mixed models and generalized estimating equations (GEE) to account for clustering and present the corresponding *P* values. Analyses will be performed using SAS (version 9.4; SAS Institute Inc).

The estimand for patient health outcomes will be the time-specific patient participant treatment effect, adjusting for clustering by primary care site. The primary outcome (LEFS) will be analyzed using linear mixed regression with restricted maximum likelihood estimates under the assumption of missing at random, which will allow the use of all available data without the need for multiple imputation. The Kenward-Rogers degrees of freedom correction will be used to account for a small number of clusters [65]. The 12-month time point will provide the intervention effect as the adjusted least square mean difference between arms with 95% CIs. Secondary comparisons will be made using adjusted least square mean differences between arms at all intermediate time points. Fixed effects in the mixed model will include time, intervention group by time interaction (omitting the group main effect), prespecified covariates associated with hip and knee function (patient participant age, sex, duration of current episode of hip or knee pain, income, highest level of education, and comorbidity score), and primary care site rurality and number of active patients (the covariates from the covariate-constrained allocation procedure). Repeated measures will be modeled using a covariance structure determined by Akaike Information Criterion and Bayesian Information Criterion. The primary care site will be included as a random effect to account for site clustering. We will assess the potential risk of bias associated with missing data by comparing the characteristics of those who participated. We plan to carry out a sensitivity analysis for a potential departure of our missing at random assumption using a delta-adjusted imputation pattern mixture model approach [66-68] to investigate the robustness of our trial outcomes with regard to the missing values of the LEFS.

We plan a secondary analysis using our primary outcome (LEFS) to compare the proportion of participants who experience a meaningful improvement in each arm (responder analysis [69]) using robust Poisson regression, accounting for clustering. We will define a meaningful change as an improvement of ≥9 points on the LEFS (the minimally important change [40,41]) and present results using relative risk. We will use empirical covariance (“sandwich”) bias-adjusted (residual-based) estimators, and apply the Fay and Graubard [70] correction for the small number of clusters.

Pain intensity (NPRS), quality of life (EQ-5D-5L), self-efficacy (PSEQ), catastrophic thinking (PCS), pain-related fear (TSK-11), and depressive symptoms (PHQ-2) will be analyzed using the same analytic approach as described for our primary LEFS analysis, adjusted for the same covariates. We will use simple mean imputation to fill in missing individual items on surveys as described by Chavance [68]. Patient satisfaction and global rating of change do not include a baseline measure and will be assessed using ordinal logistic regression with random effects and adjusting for the same covariates as above.

Incidence rates for minor adverse events (yes or no) will be calculated using robust Poisson regression and compared by calculating relative risks with CIs using GEE-type robust covariance estimators (PROC GLIMMIX, EMPIRICAL option in SAS) to account for clustering [71]. In our models comparing incidence rates, we will use an exchangeable working correlation matrix, empirical covariance (“sandwich”) bias-adjusted (residual-based) estimators, and the Fay and Graubard [70] correction to account for a small number of clusters. Given that we have only included 14 clusters and adverse events are expected to be rare, we anticipate there may be issues of nonconvergence or instability in our models. Should the model for adverse events not converge or demonstrate instability, we would attempt to fit the model using an independent working correlation matrix. If there are still issues of nonconvergence or instability, we will reduce the model by removing covariates, beginning with duration of pain and income. Severity and duration of minor adverse events will be presented descriptively. We do not anticipate many, if any, serious adverse events; as such, we plan to present these data descriptively.

Visits with primary care physicians or nurse practitioners, time (days) lost from occupational activities, and assistance required (hours) will be presented as rates and compared by calculating rate ratios using GEE-type covariance estimators with an adjusted Poisson or negative binomial model, accounting for clustering, and assuming an exchangeable working correlation matrix. Incidence rates will be used to present visits to other health professionals within the primary care team, participation in group programs offered by the primary care team, and health care services received outside of the primary care team (medications, diagnostic imaging, walk-in clinic visits, ED visits, specialist physician visits, hospital admissions, interventional procedures, surgeries, and other health provider visits). Comparisons between groups for each of these variables will be made by calculating relative risk using robust Poisson regression [71]. These models will incorporate empirical covariance (“sandwich”) bias-adjusted (residual-based) estimators, and the Fay and Graubard [70] correction due to the small number of clusters [72,73]. These models will use time as an offset to account for variable follow-up times. All of the health care use and lost time from occupational activity models will control for the same covariates as the patient health outcomes analyses and use the same approach to address cases of model nonconvergence or instability.

Our cost use analysis will be carried out from societal (primary) and health payer (secondary) perspectives. We will calculate total costs by multiplying the quantity of resource use by the corresponding unit cost, summing the total cost over each follow-up interval to determine total costs at each follow-up time point, as well as across the entire study period. Total and mean costs (overall and at each time point) will be presented by aggregated and disaggregated costs. We will estimate QALYs for every participant using the area under the curve and assuming linear interpolation between assessment time points. Bivariate multilevel modeling, accounting for clustering, will be used to analyze the incremental cost per QALY gained and describe the incremental net benefit at various values of willingness to pay. We will model the treatment group as a fixed effect and account for site clustering. We will adjust for the same covariates as the primary analyses. We will use a probabilistic sensitivity analysis with Monte Carlo simulations to explore the uncertainty in our cost-effectiveness estimates. The results will be illustrated on cost-effectiveness planes, and cost-effectiveness acceptability curves will be presented to demonstrate the likelihood that the physiotherapist-led care model is cost-effective across various willingness-to-pay thresholds.

We also plan a subgroup analysis based on sex for each of our effectiveness outcomes, as recommended in the Sex and Gender Equity in Research guidelines [33]. We will include an interaction term with sex and group, and group by time, in order to assess this. We will present the data using forest plots and 95% CIs.

##### Process Evaluation Analyses

As part of our process evaluation, the percentage of patient participants with hip or knee pain who are assessed within 48 hours of calling for an appointment will be reported descriptively and compared between arms by calculating relative risks with robust Poisson regression, using GEE-type covariance estimators to account for clustering, assuming an exchangeable working correlation matrix. Empirical covariance (“sandwich”) bias-adjusted (residual-based) estimators, and the Fay and Graubard [70] correction will be used. We will incorporate the same covariates as with our effectiveness analysis. The percentage of patient participants with hip or knee pain in the physiotherapist-led primary care arm who visited a physiotherapist as their first point of contact for the current episode of hip or knee pain will be reported descriptively.

To analyze the hip and knee pain management provided, the proportion of patient participants who receive the following will be reported descriptively: education; exercises; psychological interventions; referrals to internal primary care team members; referrals to external HCPs; medications prescribed, deprescribed, and suggested; diagnostic imaging ordered; notes to employers or insurers; and messages sent between internal primary care team members. Comparison between arms will be made using robust Poisson regression. Comparison between arms will be made using relative risks and CIs using GEE-type covariance estimators, accounting for clustering, and assuming an exchangeable working correlation matrix. Empirical covariance (“sandwich”) bias-adjusted (residual-based) estimators and the Fay and Graubard [70] correction to account for small number of clusters will be used.

Adherence to physiotherapist activity and exercise recommendations as reported at 3-month follow-up will be reported descriptively. The proportion of people accessing physiotherapist in the community will be reported descriptively and compared using robust Poisson regression, accounting for clustering.

If the intervention is effective (ie, the physiotherapist-led primary care model results in greater patient functioning than the usual care group), we will carry out mediation analyses [74] to assess potential mechanisms as part of our process evaluation. Specifically, we will assess whether changes in self-efficacy (PSEQ) or changes in psychosocial risk factors (PHQ-2, PCS, and TSK-11) explain or partially explain changes in patient functioning (LEFS score). We will conduct a separate mediation analysis for each potential variable (PSEQ, PHQ-2, PCS, and TSK-11). We will use a stepped approach proposed by Beril and colleagues [75] to investigate temporal and dynamic trends of the treatment effect across repeated measures using theoretical insights about the mediation effect to choose the appropriate mediation model. The intervention effect explained by the mediator (indirect effect) will be calculated as the difference between the total effect (the effect calculated in the primary analysis) of the physiotherapist-led primary care model on the LEFS score and the direct effect of the intervention [76,77]. We will evaluate the possibility of mediation using the significance of this effect [78,79]. We will use the LEFS outcome at the 12-month follow-up time point as the outcome variable [80,81]. This analysis will provide effect measures that allow us to report the proportion of the total effect that is mediated through each of the potential mediator variables [82]. Our causal/associated conceptual model has considered, and controlled for where needed, mediation analysis assumptions that there is no intervention-outcome, mediator-outcome, or intervention-mediator confounding or mediator-outcome confounding that is influenced by the intervention itself [77,83,84]. To explore potential mechanisms of cost differences in our cost analysis, we will report the proportion of cost differences between arms that are health care use costs and the proportion of costs associated with missed occupational activities.

In alignment with our program theory, understanding the unique experiences and perspectives of patients is a key uncertainty related to implementation of the model of care and critical to the scale and spread of the model if effective. We will explore the experiences of patient participants with hip or knee pain who have participated in the physiotherapist-led primary care model for hip or knee pain using in-depth qualitative interviews conducted and analyzed in an interpretive description tradition [62,85]. Interpretive description is a qualitative approach that is founded on naturalistic inquiry and involves focusing on identifying applied and clinically relevant themes that can inform health care or health service delivery. Interpretive description, therefore, is well-suited to helping us achieve our process evaluation goal to understand experiences and perspectives with the physiotherapist-led primary care model for people with hip or knee pain. The experiences and perspectives of patients are expected to lead to refinements in how the model of care is implemented if effective. Strategies to support rigor will include use of 2 independent coders for the first 2-3 manuscripts to ensure reliability and consistency in coding, use of reflexive journaling and reflexive dialogue among team members throughout the analytic process, incorporation of detailed field notes and written memos, long and deep engagement with the qualitative interview data, and maintaining an audit trail to document the analytic decisions throughout the research process [86-90].

#### Ethical Considerations

Ethics approval for this study has been obtained from the Queen’s University Health Science and Affiliated Teaching Hospitals Research Ethics Board (HSREB #6040471). Electronic, written, or verbal consent was obtained from all participants willing to participate.

## Results

This study was funded in December 2022 by the Arthritis Society of Canada and Canadian Institute of Health Research Stars Award (grant 22-0000000065). Our internal cluster randomized trial was registered prospectively in February 2023 (NCT05736133). Recruitment of primary care sites (clusters) was completed in June 2023 (n=14), and cluster randomization was performed in July 2023. Patient participant recruitment began in October 2023. When progression criteria for patient participant recruitment rate, assessment completion rate, and treatment fidelity were met at the 20-week time point, the decision was made to progress directly into the fully powered trial without performing any preliminary analysis of the outcome data collected during the pilot phase, with the plan to incorporate this data in the full trial analysis.

The fully powered trial was registered at ClinicalTrials.gov (NCT06358521) at the end of the pilot phase (April 2024). This registration is considered the main record for the fully powered trial. There were no changes in the outcomes collected or the timing of outcome measurement with the progression to the fully powered trial. Participant recruitment (n=647) was completed in November 2024. The last participant follow-up survey was conducted in November 2025. Extraction of data from EHRs is expected to finish by the end of December 2025. Analysis will be conducted as outlined in this analysis plan, starting after all data collection is complete, and no interim analysis was planned. Publication of results is planned for 2026.

The internal pilot for this trial was registered prospectively on February 9, 2023, at ClinicalTrials.gov (NCT05736133). When progression criteria for patient participant recruitment rate, assessment completion rate, and treatment fidelity were met at the 20-week time point, the decision was made to progress directly into the fully powered trial without performing any preliminary analysis of the outcome data collected during the pilot phase, with the plan to incorporate this data in the full trial analysis. The fully powered trial was registered on April 5, 2024, at ClinicalTrials.gov (NCT06358521).

## Discussion

### Overview

The results of this pragmatic cluster trial are expected to provide the most rigorous evidence to date on the effectiveness, cost-effectiveness, and impact of a physiotherapist-led primary care model for hip and knee pain. A key strength of the study is its comprehensive multimethods process evaluation, which will provide critical evidence to help interpret trial results and evidence to guide health system leaders and primary care teams regarding the implementation of this model if proven effective. The evaluation has been codeveloped with knowledge users—including individuals living with hip and knee pain and health care professionals engaged in the intervention—to ensure that study outcomes are meaningful and aligned with current health system priorities. By addressing gaps in existing literature that rely on small trials and low- or very low–quality evidence [29], this study’s pragmatic design significantly enhances its relevance and generalizability. The rigorous methodological descriptions and clear analysis plan augment confidence in the validity of the findings and mitigate the risk of analytical bias. Ultimately, the results are expected to directly inform the implementation of physiotherapist-led primary care models in Ontario and may have broader applicability across similar systems both nationally and internationally.

The study design has limitations that will need to be considered when interpreting the results. First, participant recruitment occurs after randomization of clinical sites, which prevents blinding of providers and may increase the risk of revealing allocation status to eligible patient participants, introducing a potential source of selection bias. Second, the study sample size was determined based on the power needed to detect an effect for the primary outcome, LEFS, but the inclusion of just 14 clusters may limit statistical power for secondary outcomes.

### Dissemination Plan

We will share our findings through peer-reviewed manuscripts on the effectiveness of the physiotherapist-led primary care model for people seeking primary care with hip or knee pain, the cost-effectiveness and health system impact of the model, how the model of care was implemented (including differences in care provided between arms), potential mechanisms (if the intervention is effective), and the experiences of patient participants. We intend to present at scientific conferences in the fields of primary care, health services, policy research, and physiotherapy. Finally, we will create tailored research summaries and policy briefs to communicate our findings with people living with pain, primary care teams, and health system decision-makers.

## Data Availability

The datasets generated or analyzed during this study are available from the corresponding author on reasonable request and with appropriate ethical approvals in place.

## Abbreviations

ED: emergency department
EHR: electronic health record
GEE: generalized estimating equation
HCP: health care provider
LEFS: Lower Extremity Functional Scale
MRC: UK Medical Research Council
NPRS: Numeric Pain Rating Scale
PCS: Pain Catastrophizing Scale
PHQ-2: 2-item Patient Health Questionnaire
PSEQ: Pain Self-Efficacy Questionnaire
QALY: quality-adjusted life year
RA: research assistant
RCT: randomized controlled trial
REDCap: Research Electronic Data Capture
SPIRIT: Standard Protocol Items: Recommendations for Interventional Trial
TSK-11: Tampa Scale of Kinesiophobia-11
